# A randomized controlled pilot trial of game-based training in individuals with spinocerebellar ataxia type 3

**DOI:** 10.1038/s41598-018-26109-w

**Published:** 2018-05-18

**Authors:** Ray-Yau Wang, Fang-Yi Huang, Bing-Wen Soong, Shih-Fong Huang, Yea-Ru Yang

**Affiliations:** 1 Department of Physical Therapy and Assistive Technology, National Yang-Ming University, Taipei, Taiwan; 2 Department of Neurology, Neurological Institute, Taipei Veterans General Hospital, Taipei, Taiwan; 3 Department of Neurology, National Yang-Ming University, Taipei, Taiwan; 4 Brain Research Center, National Yang-Ming University, Taipei, Taiwan; 5 Department of Physical Medicine and Rehabilitation, National Yang-Ming University, Taipei, Taiwan; 6 Department of Neurosurgery, Neurological Institute, Taipei Veterans General Hospital, Taipei, Taiwan; 7 Center for Neural Regeneration, Neurological Institute, Taipei Veterans General Hospital, Taipei, Taiwan

## Abstract

Exergames are interactive video games used for exercise and may have therapeutic value in people with degenerative ataxia. The purpose of this study was to investigate potential effects of exergaming training on cerebellar ataxia in people with spinocerebellar ataxia type 3 (SCA3). Nine individuals with SCA3 were recruited and randomized to either exergaming or conventional group for a 4-week training period. The severity of ataxia was measured as the primary outcome by the Scale for the Assessment and Rating of Ataxia (SARA) and by the directional control of the limit of stability test. The secondary outcomes included upper-limb function and gait performance. After training, participants in the exergaming group had a significant decrease in the total SARA score and the gait-posture SARA subscore. Participants in the conventional training group did not show a significant improvement in selected outcome measures after the 4-week training period. No significant difference was found between groups for any of these measures. Our results suggested that the exergaming training program significantly decreased ataxia. These results support implementation of exergaming training for people with SCA3.

## Introduction

Spinocerebellar ataxia (SCA) is an autosomal, dominantly inherited neurodegenerative disease with multiple subtypes. More than 30 genetic subtypes have been described. The most common subtypes are SCA type 3 (SCA3) in many countries^1,2^. SCA3 is characterized by slowly progressive gait ataxia and is often associated with truncal ataxia, limb ataxia, and dysarthria^3^. There is no effective pharmacologic treatment for decreasing the ataxia or disease progression, although, physical therapy plays an important role in controlling ataxia and improving or maintaining function through exercise training^4^. In general, physical therapy programs for degenerative cerebellar ataxia are based on intensive static and dynamic balance and coordination exercises^5^. There is some evidence that such therapeutic exercise training alleviates ataxic symptoms and improves functional activities in people with cerebellar ataxia^6–10^.

A recent systematic review suggested that the use of virtual reality tools appears to have therapeutic value in people with degenerative ataxia^5^. Exergames are video games that incorporate virtual reality and serve as an exercise tool. These video games usually involve balance and coordination challenges and help facilitate the participants’ adherence to the intervention. Exergames could present a novel, advantageous treatment tool for training individuals with SCA^10^. A previous study evaluated the effects of an 8-week balance and coordination training program (addressing ataxia, balance, and gait) using Microsoft Xbox Kinect video games in children with progressive SCA^11^. This single-group study showed that the video game-based coordinative training alleviated several signs of ataxia in adolescents with progressive ataxia^11^. Recently, we developed a balance-based exergaming program and demonstrated that it had a positive effect on postural stability in people with Parkinson’s disease^12^. In the present study, we modified that program, adding elements for coordinative training. We conducted a randomized controlled pilot trial and evaluated the effects of a 4-week program with this system, compared with a 4-week period of conventional training (12 training sessions), on ataxia in adults with SCA3. We hypothesized that those who underwent a 4-week exergaming intervention would demonstrate equal or superior performance on outcome measures compared with those who underwent conventional training.

## Results

**Figure 1:**
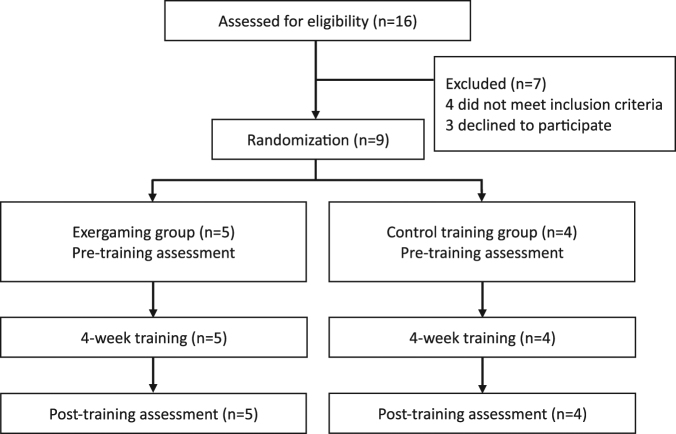
Flowchart of the experimental design.

A total of 16 individuals were screened and 9 enrolled between 2014 and 2015. Among them, 5 were assigned to the exergaming group and 4 to the control training group. All participants were assessed before and after the 4-week training period. All 9 attended all training sessions. None of the participants reported adverse events. A flow diagram of the study protocol is shown in Fig. 1.

The demographic and clinical characteristics of participants in both groups are presented in Table 1. There were no significant differences between the two groups’ demographics or clinical characteristics (established prior to the intervention) except nine-hole peg test and walking speed (Table 1).

**Table 1:** Baseline demographics and clinical characteristics of the participants (median [interval] or proportion).

Variables	Exergaming group (n = 5)	Control training group (n = 4)	*P*
Age (years)	54.0 [51.0, 60.0]	57.0 [44.0, 61.0]	1.00
Sex (male/female)	2/3	2/2	1.00
Disease duration (years)	6.0 [1.0, 16.0]	5.5 [1.0, 8.0]	0.56
Mini-mental state examination	28.0 [28.0, 29.0]	28.5 [26.0, 30.0]	1.00
More affected side (right/left)	2/3	0/4	0.44
Participant number of using walking aid (with/without)	0/5	1/3	0.44
Scale for the assessment and rating of ataxia	5.0 [3.5, 10.0]	7.5 [5.5, 13.0]	0.29
Nine-hole peg test in more affected side (sec)	26.9 [21.5, 36.2]	37.1 [32.2, 39.5]	0.03
Nine-hole peg test in less affected side (sec)	25.3 [22.9, 28.1]	31.6 [29.2, 33.8]	0.02
Walking speed (cm/sec)	112.8 [101.2, 122.7]	88.2 [26.0, 99.3]	0.02

The results of the interventions are presented in Table 2. Within-group analysis revealed that the exergaming training significantly reduced the gait-posture subscore of the Scale for the Assessment and Rating of Ataxia (SARA) (*P* = 0.038) and total SARA score (*P* = 0.042). A near-significant trend for an improvement in the limb-kinetic SARA subscore (*P* = 0.059) was also observed after exergaming training. Control training showed a marginal trend to improve the limb-kinetic SARA subscore (*P* = 0.059) and total SARA score (*P* = 0.068). There were no significant differences between the two groups for any of these measures.

**Table 2:** Outcome measures for each group (median [interval]).

Variables	Exergaming group (n = 5)			Control training group (n = 4)		
	Pre-training	Post-training	% Change	Pre-training	Post-training	% Change
Scale for the assessment and rating of ataxia						
Limb-kinetic function	2.0 [1.0, 6.0]	1.5 [1.0, 5.0]	−20.0 [−33.3, 0.0]	4.0 [3.5, 5.0]	3.3 [2.5, 4.0]	−22.5 [−28.6, −12.5]
Gait-posture	3.0 [2.0, 4.0]	2.0 [1.0, 2.0]*	−50.0 [−50.0, −33.3]	4.0 [2.0, 8.0]	2.5 [2.0, 7.0]	−22.9 [−40.0, 0.0]
Total	5.0 [3.5, 10.0]	3.5 [2.0, 7.0]*	−30.0 [−42.9, −25.0]	7.5 [5.5, 13.0]	5.8 [4.5, 11.0]	−16.8 [−33.3, −8.3]
Directional control of the limit of stability test (%)						
Forward	76.0 [63.0, 87.0]	74.0 [69.0, 83.0]	7.2 [−15.6, 14.3]	64.0 [52.0, 76.0]	68.5 [51.0, 83.0]	3.6 [−21.4, 41.4]
More affected side	79.0 [76.0, 87.0]	84.0 [73.0, 90.0]	3.4 [−10.6, 12.8]	75.0 [66.0, 89.0]	70.5 [59.0, 83.0]	−5.8 [−33.7, 25.8]
Less affected side	78.0 [74.0, 91.0]	74.0 [58.0, 83.0]	−8.1 [−26.6, 6.4]	72.5 [53.0, 86.0]	73.5 [37.0, 87.0]	−7.1 [−30.2, 20.8]
Backward	67.0 [0.0, 77.0]	56.0 [41.0, 62.0]	−15.5 [−20.8, −12.7]	23.0 [0.0, 50.0]	36.5 [0.0, 62.0]	63.9 [−100.0, 520.0]
Nine-hole peg test (sec)						
More affected side	26.9 [21.5, 36.2]	22.4 [21.6, 31.7]	−11.7 [−16.6, 2.6]	37.1 [32.2, 39.5]	37.1 [27.3, 37.9]	−2.0 [−15.1, 0.2]
Less affected side	25.3 [22.9, 28.1]	23.7 [21.8, 27.2]	−5.1 [−15.2, 4.2]	31.6 [29.2, 33.8]	28.1 [24.4, 33.0]	−9.8 [−27.7, 9.8]
Gait performance						
Walking speed (cm/sec)	112.8 [101.2, 122.7]	111.3 [99.7, 121.7]	−1.3 [−1.6, −0.6]	88.2 [26.0, 99.3]	94.9 [24.5, 112.1]	3.1 [−5.8, 21.6]
Step width (cm)	12.7 [8.3, 28.4]	15.3 [10.9, 17.3]	9.3 [−39.3, 33.1]	16.3 [14.6, 22.9]	17.1 [15.3, 21.5]	−0.6 [−8.6, 21.0]

*Is *P* ≤ 0.05 for within-group comparison.

## Discussion

This study showed that the exergaming training program significantly improved the SARA scores in people with SCA3. The exergaming training, however, did not provide better results than conventional training with respect to decreasing ataxia or improving functional performance. To our knowledge, this is the first randomized controlled trial to show that exergaming training could be adopted to improve ataxia in adults with SCA3.

The current study tested two training programs with similar training principles and protocols. Both programs consisted of balance and coordination training. We recorded average decreases in SARA scores of 1.5 and 2.0 points after conventional training and exergaming training, respectively. These results are in line with previous findings, which reported that a 4-week course of intensive coordinative training or rehabilitative training decreased SARA scores by 2.8–5.2 points^6–8^. The natural disease progression of SCA3 includes an annual increase in SARA score of 1.1–1.6 points^13–15^. These findings imply that exercise intervention may be able to postpone disease progression, especially the ataxia. Also, based on the results from those related studies, it seems that the more intensive the training, the greater is the reduction in the SARA score^10,11^.

In the current study, we recorded not only the total SARA score but also the limb-kinetic and gait-posture SARA subscores. Our results showed that only the exergaming training significantly reduced the gait-posture subscore. Limb ataxia also showed a marginal effect after the exergaming training. In the past, the beneficial effects of motor training had been challenged by the fact that the cerebellum is crucially involved in motor adaptation and motor learning^10^. It is reasonable to speculate that the Kinect sensor provides specific motor practice using motion capture, which offers precise real-time information for monitoring limb movement and guiding performance. Our findings showed that exergaming training yields a specific effect on ataxia that goes beyond mere improvement in subjects’ game scores. Therefore, people with cerebellar ataxia have enough learning capacity to benefit from exercise or motor training programs. In addition, our previous study showed that the similar exergaming training significantly improved balance in individuals with Parkinson’s disease^12^. Consistently, people with SCA3 improved more of their ataxia concerning gait and posture, as indicated by their significant improvements in gait-posture SARA subscores. The mild effect of exergaming training on the limb-kinetic subscore, however, may be due to the small sample size or short training duration. A larger sample size and longer training may support additional benefits for exergaming training.

Although SARA is the most frequently used tool to document the severity of ataxia, it does not focus on posture disorders^5^. In this study, we used the directional control during the limit of stability test to indicate trunk ataxia. It is not surprising that neither the exergaming or conventional training had a beneficial effect here as intensive rehabilitation programs with balance and coordination exercises are necessary in individuals with cerebellar ataxia^5,10^. Our previous study suggested that balance-based exergaming training using the Kinect sensor for 8 weeks resulted in positive effects on postural stability, with particularly strong effects on the directional control in people with Parkinson’s disease^12^. It has also been noted that intensive coordinative training that focuses on static and dynamic balance not only decreases the severity of ataxia but also improves gait speed and step length in people with cerebellar ataxia^11^. Gait performance, however, did not improve significantly after the exergaming or conventional training in the present study, although the severity of gait-posture ataxia decreased, especially after the exergaming training. We therefore suggest that the duration of balance training should focus on improving trunk and gait performance, especially for people with SCA3.

Of note, all participants in the exergaming group were highly motivated throughout the whole training period. None of the participants reported adverse events. Thus, incorporating technology in rehabilitation (e.g., exergaming) should be encouraged to reduce the severity of symptoms and/or improve functions. Training programs could be complemented with newly developed exergames or commercially available video games, an idea that offers a novel, advantageous training strategy for people with cerebellar ataxia.

There are several limitations in this study. First, the sample size was small, limiting the strength to interpret our results. Second, the disease was more severe among controls as indicated by nine-hole peg test and walking speed. Third, the study lacks an evaluation of a long-term period of follow-up to determine the impact on function over time, although the retention effects were proved in a previous study^7^. Larger randomized controlled clinical trials should be conducted in the future to measure functional performance after exergaming training and over the long term.

## Conclusions

This study showed that a 4-week period of exergaming training decreased ataxia in people with SCA3. The results support the potential therapeutic use of exergaming for people with SCA3. Further studies on the use of exergaming are needed to verify the clinical implications of these results.

## Methods

The Institutional Review Board of Taipei Veterans General Hospital, Taiwan, granted ethical approval for this study. The study was registered at ClinicalTrials.gov Identifier NCT02900508 on September 8, 2016.

### Study design

This study was an assessor-blinded, randomized controlled trial. The study protocol was explained to all subjects before their participation (supplementary information). Those finally participating in the study gave their consent. The study was performed in accordance with the Declaration of Helsinki. All participants were randomly assigned by block randomization to the exergaming or control training group. They learned of their assignment via a sealed envelope. Participants in the exergaming and control training groups underwent either a 4-week exergaming intervention or 4 weeks of conventional training, respectively. Measures of ataxia, upper-limb function, and gait performance were recorded before and after the training period.

### Participants

Participants were recruited from a medical center in Taipei. Information on age, sex, the more affected side, and disease duration were obtained through patient interviews and from medical charts. All participants met the following inclusion criteria: (1) genetically confirmed diagnosis of SCA3; (2) ability to walk independently with or without walking aids; (3) age ≥20 years; and (4) a score of ≥24 on the mini-mental state examination. The exclusion criteria were as follows: (1) uncontrolled medical conditions (e.g., unstable hypertension or epilepsy) and (2) a history of other neurological, cardiovascular, or orthopedic diseases that affect motor performance and balance. In total, 16 individuals were identified as potential participants for the study. Among them, 9 gave informed consent and participated in the study.

### Intervention

Participants in both groups underwent training for 40 min per session at three sessions per week for 4 weeks. Each training session began with a 5-min warm-up and ended with a 5-min cool-down. The warm-up period focused on stretching exercises of the trunk and extremities. The cool-down period focused on walking on a treadmill.

**Figure 2:**
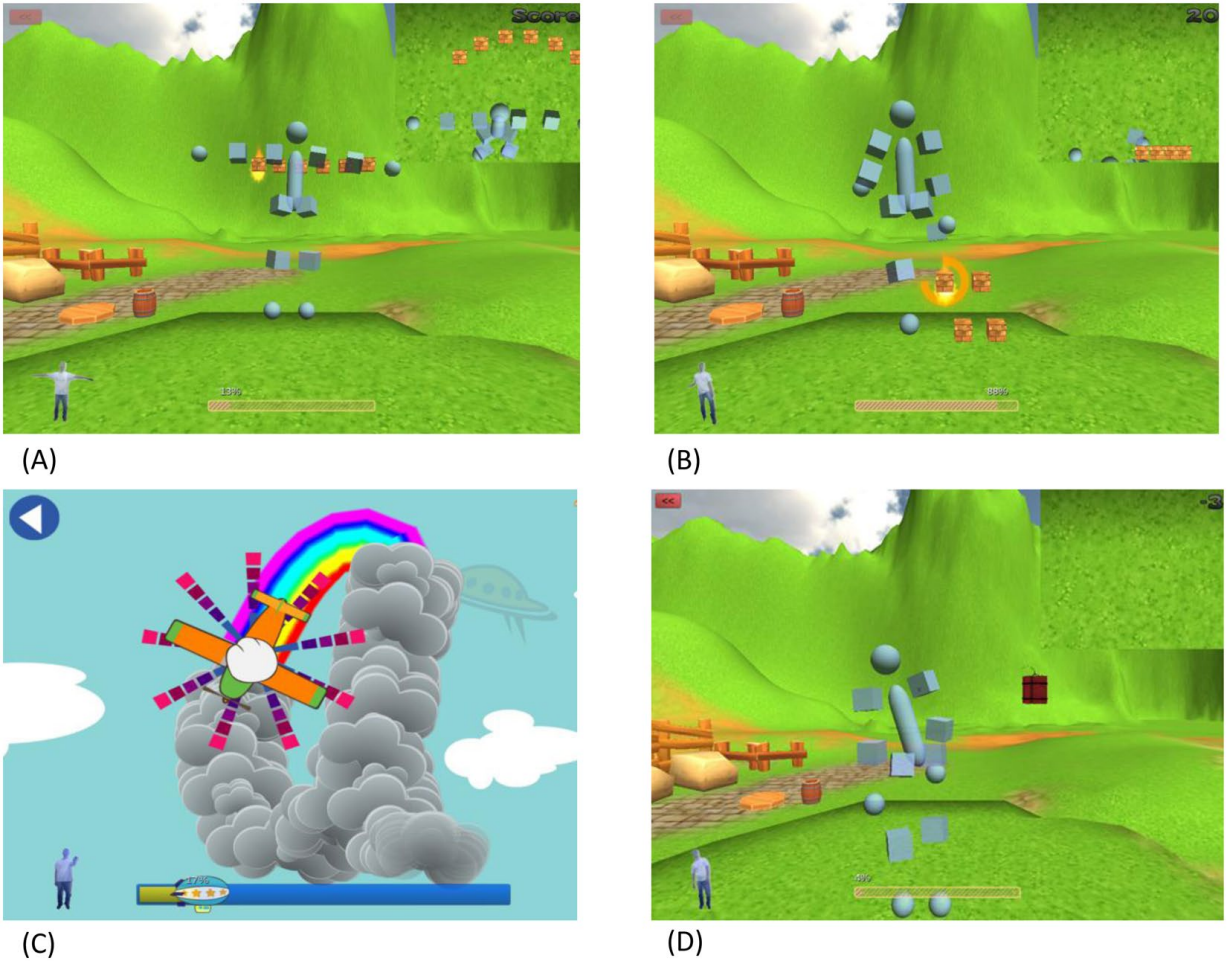
Screen shots of interaction with the exergaming program. Four exergaming programs–reaching task (**A**), pointing task (**B**), following task (**C**), avoiding task (**D**)–were designed and used for training.

Participants in the exergaming group underwent a 30-min exergaming intervention using the Kinect sensor (Microsoft Corp., Redmond, WA, USA). The Kinect sensor incorporates infrared light and a video camera, which creates a three-dimensional (3D) map of the area in front of it. This device captures full-body 3D motion. Four exergaming training programs were designed to incorporate an appropriate level of challenge to match the ability and fitness of people with SCA (Fig. 2).

- Reaching task, for balance training: Participants were asked to reach the arm toward a stationary target at a given location.
- Pointing task, for lower-limb coordination training: Participants were asked to point the foot toward a stationary target at a given location.
- Following task, for upper-limb coordination training: Participants were asked to track an airplane with a hand as the airplane flew in 3D space.
- Avoiding task, for trunk coordination training: Participants were instructed to avoid upcoming obstacles that approached from varying directions at random by moving the body right/left or up/down.

During the training duration, the challenge level was increased progressively by adjusting the posture (sitting, kneeling, half-kneeling, standing), changing the base of support, amplitude, frequency, speed, complexity, and number of hints. The details of the exergaming programs are shown in Table 3.

**Table 3:** Exergaming intervention program.

Program	Action	Progression	Motor demand
Reaching task	Reaching by arm toward a stationary target at different heights, depths and in different directions	• Reaching length • Number of targets • Range of distribution • Amount of repetition • Different action positions	• Weight shifting • Challenging balance • Functional transitions
Pointing task	Pointing by foot toward a stationary target at different heights, depths and in different directions	• Number of targets • Range of distribution • Amount of repetition • Different action positions	• Leg coordination • Advance motor planning
Following task	Tracking an airplane by hand as the airplane flew in 3D space	• Speed • Moving range • Remembered sequence or course of trajectory • Different action positions	• Arm coordination • Advance motor planning
Avoiding task	Avoiding the upcoming obstacles that randomly approached from varying directions by moving body sideways or up/down	• Hitting direction • Speed • Obstacle hitting ratio • Different action positions	• Trunk coordination • Movement adaption • Agility

Participants in the control training group underwent a 30-min conventional balance and coordination training session. The training program included reaching activities as well as upper-limb, lower-limb, and trunk coordination activities. The general training protocols used for the control training group were the same as those used for the exergaming group except for capturing the 3D motion. The challenge level was increased progressively by adjusting the posture and changing the base of support, speed, and complexity (Table 4).

**Table 4:** Control intervention program.

Program	Action	Progression	Motor demand
Reaching task	Reaching by arm toward a stationary cup at different heights, depths and in different directions	• Reaching length • Number of targets • Range of distribution • Amount of repetition • Different action positions	• Weight shifting • Challenging balance • Functional transitions
Tossing task	Tossing by foot to a stationary cone at different heights, depths and in different directions	• Number of targets • Range of distribution • Amount of repetition • Different action positions	• Leg coordination • Advance motor planning
Following task	Tracking (following) the therapist’s hand by participant’s hand as the therapist’s hand moved in different directions	• Speed • Moving range • Remembered sequence or course of trajectory • Different action positions	• Arm coordination • Advance motor planning
Whole body task	Performing the trunk movements with upper and lower extremity movements in a given rhythm and command	• Speed • Range of distribution • Different action positions	• Trunk coordination • Movement adaption • Agility

### Outcome measures

#### Ataxia

The primary outcome measure for ataxia was the Scale for the Assessment and Rating of Ataxia. This scale, which consists of eight items, is a valid, highly reliable tool for evaluating SCA^16–18^. Possible total scores using the SARA range from 0 to 40 points, with a higher score indicating more severe ataxia. Four of the eight items in the SARA involve limb-kinetic function (finger chase, nose–finger test, fast alternating hand movements, heel-shin slide) and three involve gait and posture (gait, stance, sitting)^19,20^. We calculated the limb-kinetic subscore from the sum of finger chase, nose–finger test, fast alternating hand movements, and heel-shin slide and the gait-posture subscore from the sum of gait, stance, and sitting.

The directional control of the limit of stability test by the Smart Balance Master (NeuroCom International Inc., Clackamas, OR, USA) was first used to indicate possible trunk ataxia. The directional control is defined as the amount of movement in the intended direction minus the amount of extraneous movement. A directional control score of 100% indicates that the participant does not deviate from a straight path during the test. Therefore, the directional control was used to indicate possible trunk ataxia quantitatively during balance control in the standing position in this study. To assess the limit of stability, participants stood on a force plate and shifted their center of gravity to reach a maximum distance in the target direction as quickly and accurately as possible without moving their feet. The directions assessed included forward, less affected side, more affected side, and backward, in random order.

#### Upper-limb function

The nine-hole peg test was used to assess upper-limb function. During the test, participants picked up the pegs one at a time and put them into the holes, in any order, until the holes were filled. They then removed the pegs one at a time and returned them to the container. The time needed to complete the test was recorded. Both upper limbs were tested twice, and the mean of the two tests for each limb was calculated. The more affected and less affected sides were identified based on the time needed to complete the test.

#### Gait performance

The GAITRite system (GAITRite, CIR Systems Inc., Franklin, NJ, USA) was used to evaluate gait performance. It comprises a portable carpet walkway (length 5 m, width 0.9 m) with 16,128 embedded sensors along its length. The sampling rate of the system is 80 Hz. When the participant walks on the carpet, the sensors under the carpet collect data on spatial and temporal gait parameters. Participants were instructed to walk at a comfortable pace for five trials. The collected data were then averaged. Gait parameters of interest were velocity (cm/sec) and step width (cm).

#### Statistical analysis

All analyses were performed using the SPSS 20.0 statistical package (SPSS Inc., Chicago, IL, USA). The distributions of the variables were expressed as the medians and interval. The general characteristics of two groups were compared using the *χ*^2^ and Mann-Whitney U test for categorical and continuous variables, respectively. The Wilcoxon signed-rank test was performed for within-group comparisons. To adjust for between-group baseline differences, percentage changes of the variables were calculated and analyzed by the Mann-Whitney U test for between-group comparisons. Statistical significance was set at *P* ≤ 0.05.

## Electronic supplementary material

CONSORT

Clinical trial protocol
