# Supplementary material for: A randomized controlled pilot trial of game-based training in individuals with spinocerebellar ataxia type 3
Source: Sci Rep. 2018 May 18;8:7816. doi: 10.1038/s41598-018-26109-w (PMC5959926; doi:10.1038/s41598-018-26109-w)
Supplement: Supplementary file 2 — Clinical trial protocol [file 41598_2018_26109_MOESM2_ESM.docx]

**Trial Protocol**

**General information**

**Protocol title:** A randomized controlled pilot trial of game-based training in individuals with spinocerebellar ataxia type 3

**Protocol identifying number and date:** NCT02900508 and September 8, 2016

**Name and address of the funder:** National Science Council, Taiwan

**Name and title of the investigators** who are responsible for conducting the research, and the address and telephone numbers of the research sites including responsibilities of each:

Ray-Yau Wang^1^, Fang-Yi Huang^1^, Bing-Wen Soong^2,3,4^, Shih-Fong Huang^5,6,7^ & Yea-Ru Yang^1^

^1^Department of Physical Therapy and Assistive Technology, National Yang-Ming University, Taipei, Taiwan. ^2^Department of Neurology, Neurological Institute, Taipei Veterans General Hospital, Taipei, Taiwan. ^3^Department of Neurology, National Yang-Ming University, Taipei, Taiwan. ^4^Brain Research Center, National Yang-Ming University, Taipei, Taiwan. ^5^Department of Physical Medicine and Rehabilitation, National Yang-Ming University, Taipei, Taiwan. ^6^Department of Neurosurgery, Neurological Institute, Taipei Veterans General Hospital, Taipei, Taiwan. ^7^Center for Neural Regeneration, Neurological Institute, Taipei Veterans General Hospital, Taipei, Taiwan.

**Project summary**

Exergames are interactive video games used for exercise and may have therapeutic value in people with degenerative ataxia. The purpose of this study was to investigate potential effects of exergaming training on cerebellar ataxia in people with spinocerebellar ataxia type 3 (SCA3). Nine individuals with SCA3 were recruited and randomized to either exergaming or conventional group for a 4-week training period. The severity of ataxia was measured as the primary outcome by the Scale for the Assessment and Rating of Ataxia (SARA) and by the directional control of the limit of stability test. The secondary outcomes included upper-limb function and gait performance. After training, participants in the exergaming group had a significant decrease in the total SARA score and the gait-posture SARA subscore. Participants in the conventional training group did not show a significant improvement in selected outcome measures after the 4-week training period. No significant difference was found between groups for any of these measures. Our results suggested that the exergaming training program significantly decreased ataxia. These results support implementation of exergaming training for people with SCA3.

**Rationale & background information**

Spinocerebellar ataxia (SCA) is an autosomal, dominantly inherited neurodegenerative disease with multiple subtypes. More than 30 genetic subtypes have been described. The most common subtypes are SCA type 3 (SCA3) in many countries[^1^](#_ENREF_1)^,^ [^2^](#_ENREF_2). SCA3 is characterized by slowly progressive gait ataxia and is often associated with truncal ataxia, limb ataxia, and dysarthria[^3^](#_ENREF_3). There is no effective pharmacologic treatment for decreasing the ataxia or disease progression, although, physical therapy plays an important role in controlling ataxia and improving or maintaining function through exercise training[^4^](#_ENREF_4). In general, physical therapy programs for degenerative cerebellar ataxia are based on intensive static and dynamic balance and coordination exercises[^5^](#_ENREF_5). There is some evidence that such therapeutic exercise training alleviates ataxic symptoms and improves functional activities in people with cerebellar ataxia[^6-10^](#_ENREF_6).

A recent systematic review suggested that the use of virtual reality tools appears to have therapeutic value in people with degenerative ataxia[^5^](#_ENREF_5). Exergames are video games that incorporate virtual reality and serve as an exercise tool. These video games usually involve balance and coordination challenges and help facilitate the participants’ adherence to the intervention. Exergames could present a novel, advantageous treatment tool for training individuals with SCA[^10^](#_ENREF_10). A previous study evaluated the effects of an 8-week balance and coordination training program (addressing ataxia, balance, and gait) using Microsoft Xbox Kinect video games in children with progressive SCA[^11^](#_ENREF_11). This single-group study showed that the video game-based coordinative training alleviated several signs of ataxia in adolescents with progressive ataxia[^11^](#_ENREF_11). Recently, we developed a balance-based exergaming program and demonstrated that it had a positive effect on postural stability in people with Parkinson’s disease[^12^](#_ENREF_12). In the present study, we modified that program, adding elements for coordinative training. We conducted a randomized controlled pilot trial and evaluated the effects of a 4-week program with this system, compared with a 4-week period of conventional training (12 training sessions), on ataxia in adults with SCA3. We hypothesized that those who underwent a 4-week exergaming intervention would demonstrate equal or superior performance on outcome measures compared with those who underwent conventional training.

**Study goals and objectives**

The purpose of this study was to investigate potential effects of exergaming training on cerebellar ataxia in people with SCA3.

**Study Design**

This study was an assessor-blinded, randomized controlled trial. The study protocol was explained to all subjects before their participation. Those finally participating in the study gave their consent. The study was performed in accordance with the Declaration of Helsinki. All participants were randomly assigned by block randomization to the exergaming or control training group. They learned of their assignment via a sealed envelope. Participants in the exergaming and control training groups underwent either a 4-week exergaming intervention or 4 weeks of conventional training, respectively. Measures of ataxia, upper-limb function, and gait performance were recorded before and after the training period.

**Methodology**

The Institutional Review Board of Taipei Veterans General Hospital, Taiwan, granted ethical approval for this study. The study was registered at ClinicalTrials.gov Identifier NCT02900508 on September 8, 2016.

*Participants*

Participants were recruited from a medical center in Taipei. Information on age, sex, the more affected side, and disease duration were obtained through patient interviews and from medical charts. All participants met the following inclusion criteria: (1) genetically confirmed diagnosis of SCA3; (2) ability to walk independently with or without walking aids; (3) age ≥ 20 years; and (4) a score of ≥ 24 on the mini-mental state examination. The exclusion criteria were as follows: (1) uncontrolled medical conditions (e.g., unstable hypertension or epilepsy) and (2) a history of other neurological, cardiovascular, or orthopedic diseases that affect motor performance and balance. In total, 16 individuals were identified as potential participants for the study. Among them, 9 gave informed consent and participated in the study.

*Intervention*

Participants in both groups underwent training for 40 min per session at three sessions per week for 4 weeks. Each training session began with a 5-min warm-up and ended with a 5-min cool-down. The warm-up period focused on stretching exercises of the trunk and extremities. The cool-down period focused on walking on a treadmill.

Participants in the exergaming group underwent a 30-min exergaming intervention using the Kinect sensor (Microsoft Corp., Redmond, WA, USA). The Kinect sensor incorporates infrared light and a video camera, which creates a three-dimensional (3D) map of the area in front of it. This device captures full-body 3D motion. Four exergaming training programs were designed to incorporate an appropriate level of challenge to match the ability and fitness of people with SCA (Figure 2).

- Reaching task, for balance training: Participants were asked to reach the arm toward a stationary target at a given location.
- Pointing task, for lower-limb coordination training: Participants were asked to point the foot toward a stationary target at a given location.
- Following task, for upper-limb coordination training: Participants were asked to track an airplane with a hand as the airplane flew in 3D space.
- Avoiding task, for trunk coordination training: Participants were instructed to avoid upcoming obstacles that approached from varying directions at random by moving the body right/left or up/down.

During the training duration, the challenge level was increased progressively by adjusting the posture (sitting, kneeling, half-kneeling, standing), changing the base of support, amplitude, frequency, speed, complexity, and number of hints. The details of the exergaming programs are shown in Table 3.

Participants in the control training group underwent a 30-min conventional balance and coordination training session. The training program included reaching activities as well as upper-limb, lower-limb, and trunk coordination activities. The general training protocols used for the control training group were the same as those used for the exergaming group except for capturing the 3D motion. The challenge level was increased progressively by adjusting the posture and changing the base of support, speed, and complexity (Table 4).

*Outcome measures*

*Ataxia*

The primary outcome measure for ataxia was the Scale for the Assessment and Rating of Ataxia. This scale, which consists of eight items, is a valid, highly reliable tool for evaluating SCA[^13-15^](#_ENREF_13). Possible total scores using the SARA range from 0 to 40 points, with a higher score indicating more severe ataxia. Four of the eight items in the SARA involve limb-kinetic function (finger chase, nose–finger test, fast alternating hand movements, heel-shin slide) and three involve gait and posture (gait, stance, sitting)[^16^](#_ENREF_16)^,^ [^17^](#_ENREF_17). We calculated the limb-kinetic subscore from the sum of finger chase, nose–finger test, fast alternating hand movements, and heel-shin slide and the gait-posture subscore from the sum of gait, stance, and sitting.

The directional control of the limit of stability test by the Smart Balance Master (NeuroCom International Inc., Clackamas, OR, USA) was first used to indicate possible trunk ataxia. The directional control is defined as the amount of movement in the intended direction minus the amount of extraneous movement. A directional control score of 100% indicates that the participant does not deviate from a straight path during the test. Therefore, the directional control was used to indicate possible trunk ataxia quantitatively during balance control in the standing position in this study. To assess the limit of stability, participants stood on a force plate and shifted their center of gravity to reach a maximum distance in the target direction as quickly and accurately as possible without moving their feet. The directions assessed included forward, less affected side, more affected side, and backward, in random order.

*Upper-limb function*

The nine-hole peg test was used to assess upper-limb function. During the test, participants picked up the pegs one at a time and put them into the holes, in any order, until the holes were filled. They then removed the pegs one at a time and returned them to the container. The time needed to complete the test was recorded. Both upper limbs were tested twice, and the mean of the two tests for each limb was calculated. The more affected and less affected sides were identified based on the time needed to complete the test.

*Gait performance*

The GAITRite system (GAITRite, CIR Systems Inc., Franklin, NJ, USA) was used to evaluate gait performance. It comprises a portable carpet walkway (length 5 m, width 0.9 m) with 16,128 embedded sensors along its length. The sampling rate of the system is 80 Hz. When the participant walks on the carpet, the sensors under the carpet collect data on spatial and temporal gait parameters. Participants were instructed to walk at a comfortable pace for five trials. The collected data were then averaged. Gait parameters of interest were velocity (cm/sec) and step width (cm).

**Data Management and Statistical Analysis**

All analyses were performed using the SPSS 20.0 statistical package (SPSS Inc., Chicago, IL, USA). The distributions of the variables were expressed as the medians and interval. The general characteristics of two groups were compared using the 𝜒^2^ and Mann-Whitney U test for categorical and continuous variables, respectively. The Wilcoxon signed-rank test was performed for within-group comparisons. To adjust for between-group baseline differences, percentage changes of the variables were calculated and analyzed by the Mann-Whitney U test for between-group comparisons. Statistical significance was set at *P* ≤ 0.05.

**Expected Outcomes of the Study**

We expected that those who underwent a 4-week exergaming intervention would demonstrate equal or superior performance on outcome measures compared with those who underwent conventional training.

**Project Management**

This study was designed by R.Y.W., F.Y.H and Y.R.Y; data were collected by F.Y.H.; data interpretation was undertaken by all authors; the manuscript was written by R.Y.W. and Y.R.Y and was proofread by all authors. All authors have approved the final version of the paper.

**Ethics**

The study was registered at ClinicalTrials.gov Identifier NCT02900508 on September 8, 2016. The study protocol was explained to all subjects before their participation. Those finally participating in the study gave their consent. The study was performed in accordance with the Declaration of Helsinki.

**Budget**

This work was supported by the National Science Council (grant number NSC100-2314-B-010-021-MY2).

**References**

1. Paulson, H. L. Dominantly inherited ataxias: Lessons learned from Machado-Joseph disease/spinocerebellar ataxia type 3. *Semin Neurol*. **27**, 133-142 (2007).

2. Pedroso, J. L. *et al*. Nonmotor and extracerebellar features in Machado-Joseph disease: A review. *Mov Disord*. **28**, 1200-1208, doi: 10.1002/mds.25513 (2013).

3. Rüb, U. *et al*. Spinocerebellar ataxia type 3 (Machado–Joseph disease): Severe destruction of the lateral reticular nucleus. *Brain*. **125**, 2115-2124 (2002).

4. Zesiewicz, T. A. *et al*. Comprehensive systematic review summary: Treatment of cerebellar motor dysfunction and ataxia: Report of the Guideline Development, Dissemination, and Implementation Subcommittee of the American Academy of Neurology. *Neurology*. doi: 10.1212/WNL.0000000000005055 (2018).

5. Marquer, A., Barbieri, G. & Perennou, D. The assessment and treatment of postural disorders in cerebellar ataxia: A systematic review. *Ann Phys Rehabil Med*. **57**, 67-78, doi: 10.1016/j.rehab.2014.01.002 (2014).

6. Ilg, W. *et al*. Intensive coordinative training improves motor performance in degenerative cerebellar disease. *Neurology*. **73**, 1823-1830, doi: 10.1212/WNL.0b013e3181c33adf (2009).

7. Ilg, W. *et al*. Long-term effects of coordinative training in degenerative cerebellar disease. *Mov Disord*. **25**, 2239-2246, doi: 10.1002/mds.23222 (2010).

8. Miyai, I. *et al*. Cerebellar ataxia rehabilitation trial in degenerative cerebellar diseases. *Neurorehabil Neural Repair*. **26**, 515-522, doi: 10.1177/1545968311425918 (2012).

9. Chang, Y. J. *et al*. Cycling regimen induces spinal circuitry plasticity and improves leg muscle coordination in individuals with spinocerebellar ataxia. *Arch Phys Med Rehabil*. **96**, 1006-1013, doi: 10.1016/j.apmr.2015.01.021 (2015).

10. Synofzik, M. & Ilg, W. Motor training in degenerative spinocerebellar disease: ataxia-specific improvements by intensive physiotherapy and exergames. *BioMed Res Int*. **2014**, 583507, doi: 10.1155/2014/583507 (2014).

11. Ilg, W. *et al*. Video game-based coordinative training improves ataxia in children with degenerative ataxia. *Neurology*. **79**, 2056-2060, doi: 10.1212/WNL.0b013e3182749e67 (2012).

12. Shih, M. C., Wang, R. Y., Cheng, S. J. & Yang, Y. R. Effects of a balance-based exergaming intervention using the kinect sensor on posture stability in individuals with parkinson's disease: a single-blinded randomized controlled trial. *J Neuroeng Rehabil*. **13**, 78, doi: 10.1186/s12984-016-0185-y (2016).

13. Subramony, S. H. SARA--a new clinical scale for the assessment and rating of ataxia. *Nat Clin Pract Neurol*. **3**, 136-137 (2007).

14. Weyer, A. *et al*. Reliability and validity of the scale for the assessment and rating of ataxia: a study in 64 ataxia patients. *Mov Disord*. **22**, 1633-1637 (2007).

15. Schmitz-Hubsch, T. *et al*. Responsiveness of different rating instruments in spinocerebellar ataxia patients. *Neurology*. **74**, 678-684, doi: 10.1212/WNL.0b013e3181d1a6c9 (2010).

16. Schmitz-Hubsch, T. *et al*. Scale for the assessment and rating of ataxia: Development of a new clinical scale. *Neurology*. **66**, 1717-1720 (2006).

17. Lawerman, T. F. *et al*. Construct validity and reliability of the SARA gait and posture sub-scale in early onset ataxia. *Front Hum Neurosci*. **11**, 605, doi: 10.3389/fnhum.2017.00605. eCollection 2017 (2017).
